# Legal recommendations and psychological advice sports administrators can implement in the next pandemic

**DOI:** 10.1007/s40318-023-00240-x

**Published:** 2023-06-12

**Authors:** Salim Yaacoub, Adrian McInman

**Affiliations:** 1 Assistant Professor, College of Law, Qatar University, Doha, Qatar; 2 Sport Psychologist, Phenomenal Performances, New York, USA

**Keywords:** Law sport, Sport psychology, Bio-bubbles, Pandemic, COVID-19, Athletes

## Abstract

The COVID-19 pandemic exposed sports administrators’ lack of crisis management preparedness and athletes, coaches, and support staff’s lack of mental preparedness for a pandemic. Sports administrators were in the unenviable position of having to protect the health of their athletes, staff, and the wider population by preventing the spread of the COVID-19 disease with restrictive policies, such as bio-bubbles and quarantines, while at the same time not restricting anyone’s liberty and unalienable rights to pursue happiness. This article informs sports administrators how to balance health instructions from regulatory bodies with measures protecting individual liberty. It provides sports administrator’s legal recommendations they can follow and psychological advice that they can pass on to their athletes, coaches, and support staff. The article also explains to coaches and support staff how to manage, and athletes how to successfully cope with, future bio-bubbles, quarantines, and the next pandemic.

## Introduction

The global COVID-19 pandemic wreaked havoc on an unprecedented scale. As per February 2023, there have been more than 750 million confirmed cases of COVID-19, including more than 680 million deaths, reported to the World Health Organization.^1^ Cambridge University estimates the cost to the global economy will be US$82 trillion over 5 years.^2^ In many countries, the devastation has already begun to leave long-term scars and the pandemic-driven lockdowns “will constitute a cultural legacy which will live long in our memories and those of future generations”.^3^

In some countries, every aspect of society was impacted. For instance, the pandemic had a profound impact on agriculture, the food supply chain, and food security^4^; businesses, such as construction, manufacturing, and retail industries^5^; corporate social responsability, consumer ethics, and basic marketing philosophy^6^; research^7^; teaching and learning^8^; and waste management.^9^ The sports industry was no exception. Many sporting events in 2020 and 2021 were canceled due to fear of contamination. Perhaps the most high-profile impact was the 12-month postponement of the Olympic Games.^10^ Likewise, the Super Rugby competition,^11^ the British Open,^12^ and the All England Club’s Wimbledon tennis Championships were canceled.^13^ In the United States, the National Basketball Association suspended its season for many months before forming a bio-secure hub in Florida where the remaining games were played without crowds^14^ and Major League Baseball delayed its season for 4 months.^15^ Champions League, Europa League, Bundesliga,^16^ Premier League,^17^ and Lega Calcio^18^ football matches were all suspended until further notice.^19^ This decision caused widespread disruption across European football. For instance, Real Madrid was placed into quarantine, which led to the suspension of LaLiga.^20^ In Australia, both the Australian Football League and the National Rugby League suspended their just-started 2020 competitions before a restart, initially with no crowds, and later with restricted numbers.^21^

In Taiwan, due to the Super Basketball League only having five teams and the country successfully containing the virus, the competition was able to go ahead. Nevertheless, a substantial number of procedures were implemented, such as forehead temperature checks, outlawing high-fives, encouraging spraying rubbing alcohol on players’ hands during timeouts, handwashing at halftime and after the match, not utilizing fans, and no spectators were in attendance.^22^ In other parts of the world, however, tournaments and international sports were only able to resume with the implementation of bio-bubbles designed to ensure the safety of players, support staff, and all people involved in the organization of the event. The bio-bubble protocol involved substantial isolation from the outside world and a large number of steps.^23^ Before departure, players, coaches, and support staff were tested for COVID-19. Upon arrival, oftentimes via a purposefully chartered flight, they were tested again. They were then subject to quarantine for 5–14 days. During the actual competitions, they usually stayed in an isolated environment where their movement was severely restricted to just a hotel and the training and competition sports venues. Anyone testing positive or developing COVID-19-related symptoms was placed in a more restrictive quarantine zone with additional medical procedures.

Many athletes had difficulties adjusting to bubble life. For instance, tennis player Alexander Zverev told the media, “In Rotterdam I was freaking out. We only stay at the hotel and on Court. There is no fresh air to breathe. We have no contact with the outside world”.^24^ Likewise, English cricketer Jofra Archer likened bubble life to imprisonment, stating in the bubbles he was “just counting the days down until [I was] free again”.^25^

Most studies assessing the psychological impact of quarantine reported dysfunctional psychological effects, such as anger, confusion, and post-traumatic stress symptoms.^26^ The following year, attitudes toward COVID-19 and mental health measurements of 56,679 adults from 34 provinces in China were assessed.^27^ They discovered quarantine was associated with an increased risk of experiencing mental health issues. They also noted that individuals with pre-existing mental disorders, pre-existing chronic physical diseases, frontline workers, those in the most severely affected areas during the outbreak, infected or suspected patients, and less financially well-off individuals were even more likely to experience mental health issues. Similar results were observed with Canadian health care workers quarantined because of exposure to SARS. They expressed a wide range of emotions including anger, fear, frustration, and lack of control.^28^ Disturbingly, long-term negative psychological effects of quarantine were found in a study of 549 individuals who lived in Beijing during the 2003 SARS outbreak. The researchers discovered 60% (29 of 48) of those with high levels of depression in 2006 had been quarantined in 2003 where: “the experience of being quarantined can… lead to long-term adverse mental health consequences”.^29^

A study that evaluated media reports published between 1st July 2020 and 31st May 2021, found eight cases of athletes withdrawing from a sporting competition due to bio-bubble fatigue.^30^ The authors warned that spending a lot of time alone could lead to adverse mental health effects, especially rumination, and proposed, “every team must have a sports medicine physician/sports psychologist at all times during the tournaments to make sure that the players remain mentally fit during this phase of COVID-19 pandemic”.^31^ Making the situation more problematic, many athletes and staff ended up traveling from one bio-bubble to the next with no break from such restricted movement. South African cricket captain Quinton de Kock is a good example of an athlete pulling out of a sporting event due to the perceived adverse effects of bubble life. He stated he was feeling the strain of living in the confined spaces of bio-secure bubbles, which he described as “unsettling,” and questioned the sustainability of “bubble life”.^32^ Likewise, English cricketer, Ben Stokes took an “indefinite break from cricket with immediate effect ‘to prioritize his mental wellbeing’”.^33^ The Managing Director of England men’s cricket, Ashley Giles, claimed: “Spending significant amounts of time away from family, with minimal freedoms, is extremely challenging. The cumulative effect of operating almost continuously in these environments has had a major impact on everyone”.^34^

Other athletes succumbed to bubble fatigue. For instance, English cricketer Mark Wood withdrew from the Indian Premier League auction to be able to spend time with his family; Josh Hazelwood and Mitchell Marsh made themselves unavailable due to bubble fatigue; Tom Banton and Tom Curran pulled out of Australia’s Big Bash League; and Kieron Pollard, David Warner, and Andre Russell withdrew from the first season of the England and Wales Cricket Board’s The Hundred Tournament.^35^ The President of the Australian Cricketers’ Association, Shane Watson, stated bubble life has “complicated every aspect” of a cricketer’s life, the extended periods away from loved ones caused a “zero life balance,” and that bubble fatigue could shorten careers: “If we don’t do something about this as time goes on, we might lose a number of our best cricketers”.^36^ Likewise, a Pakistan Cricket Board press release explained that part of the reason why both head coach Misbah-ul-Haq and bowling coach Waqar Younis unexpectedly resigned after completing only 2 of their 3-year contracts was bubble fatigue.^37^ Misbah-ul-Haq noted, that he had just finished a bio-bubble in Jamaica, was not “in the right frame of mind for the upcoming challenges,” and didn’t want to “continue to spend considerable time away from my family in a bio-secure environment”.^38^

The COVID-19 pandemic created opposing difficulties for sports administrators to manage. On the one hand, they had to create and implement policies that prevented the spread of the disease and follow instructions from other regulatory bodies, such as international sporting organizations, appropriate countries’ Ministries of Health, the Centres for Disease Control and Prevention, and the World Health Organization. On the other hand, they have to pay attention to desires of individual liberty, expressed in documents such as the International Declaration of Human Rights, the European Convention of Human Rights, and the Declaration on the Rights of the Child. Finding the right balance between preventing the spread of the disease (often with little time to make decisions) and the continued respect for human rights is problematic. English tennis player Johanna Konta believed athletes understood the need for such a balancing act, but warned there was also a need for “giving space to flexibility, to start giving us a little bit of normality”.^39^

The pandemic exposed sports administrators’ lack of preparedness for a pandemic and a subsequent need to revisit and improve crisis management strategies. The world has endured a variety of pandemics and thus Charlie Campbell, Yuxi Yunnan and Alice Park are correct when they suggest we need to be prepared “for the inevitable next time”.^40^ This article provides sports administrators with comprehensive legal recommendations and psychological advice they can implement to help their athletes, coaches, and support staff cope more effectively with future bio-bubbles and quarantines, and the next pandemic.

## Legal requirements for sports administrators, coaches, support staff, and athletes

Unless athletes and support staff follow established rules and guidelines for their respective sport, they risk being subject to sanctions. It is incumbent upon appropriate sport administrators to assist their athletes and staff follow national, state and local regulations. In so doing, sports administrators must avoid violating any of the athlete’s and/or staff’s human rights. However, international human rights treaties generally do not impose direct legal obligations on private actors. Instead, states are responsible for enacting and enforcing national legislation that can have the effect of requiring local entities to respect human rights.^41^ When creating and implementing policies that prevent the spread of COVID-19, sports administrators need to take into consideration the recommendations and instructions from other regulatory bodies, such as appropriate country’s Ministries of Health, the Centres for Disease Control and Prevention, the World Health Organization, and where relevant governing international sporting organizations. At the same time, sports administrators need to ensure that the policies they create or follow to protect the health of their athletes, support staff, and the wider population, do not inadvertently breach the International Covenant on Economic, Social and Cultural Rights (ICESCR) requirements. By respecting and following ICESCR protocols, sports administrators will also act as role models for their organization and athletes. Bearing in mind that there have been numerous examples of coaches breaching bio-bubble protocols, this exemplar behavior cannot be undervalued. For instance, Ben Hart, assistant coach of the Australian Football League’s (AFL) Adelaide Crows team, did not ensure that players trained in pairs, as they were supposed to, but trained in two groups of eight.^42^ Although police chose not to fine him or the club, the AFL suspended him for 4 weeks. Likewise, Nathan Buckley, Collingwood’s AFL Head Coach, and his assistant, Brenton Sanderson, were not mentorship material, as they were fined a total of AUD $50,000 for leaving their hotel to play a game of tennis with someone outside the club’s bio-bubble.^43^

To help sports administrators avoid violating any human rights, because they are influenced in their interpretation by the wider contextual moral, political, international and legal bubble,^44^ we will provide guidance about five concerns that sports administrators should take into consideration. These concerns are related to the right to private and family life, adequate standard of living, health, food, and the respect of their legal duties toward the athletes.

### Encourage relationship development to conserve the right to private and family life

The right to life is protected and well established in major international and regional frameworks, such as Article 3 of the Universal Declaration of Human Rights,^45^ Article 2 of the European Convention on Human Rights^46^ (ECHR 1950), Article 3 of the International Covenant on Civil and Political Rights,^47^ Article 4 of the American Convention on Human Rights,^48^ and Article 4 of the African Charter on Human and People’s Rights.^49^ It has been referred to as the “first right of man”^50^ and the most fundamental of all rights^51^ where no exemption is permitted, even in public emergencies.^52^ Hence, the bio-bubble environment and restrictions should not violate this fundamental right or other human rights. For instance, even in situations like when the Director-General of the World Health Organization, following the recommendations of the Emergency Committee, declared the outbreak on 30 January 2020 a Public Health Emergency of International Concern (PHEIC)^53^ there should not be any exemption.

The COVID-19 pandemic posed potential infringements to the right of citizens to privacy and other qualified rights. To prevent spreading the pandemic, some states collected citizen’s medical data, information about whether they were complying with quarantine rules, and who infected people had been in contact with. For instance, in Italy, phone geolocation data was used to track individual movements, in Singapore, a government-sponsored smartphone app (TraceTogether) was used for contact tracing, and in South Korea and Taiwan, individual body temperatures were monitored in public spaces, and in China red, yellow, or green codes were assigned to citizens, depending on COVID-19 test results, that either entitled or barred citizens from using public transportation or resume work.^54^ Under these conditions, a State may interfere with the enjoyment of a protected right, set out in paragraph 2 of Article 8 (right to respect for private and family life and home), namely in the interests of national security, public safety or the economic wellbeing of the country, for the prevention of disorder or crime, for the protection of health or morals, or for the protection of the rights and freedoms of others.

This is a complex issue, especially as some actions (e.g., monitoring an individual’s temperature that you might think is being proactive) can be considered a breach of article 8 of the ECHR,^55^ while other actions, such as “requesting targeted group of sports professionals to notify their precise locations in a daily time slot of 60 min for the purposes of unannounced anti-doping tests” are usually viewed as a breach. The latter is the case of Fédération Nationale des Syndicats Sportifs (FNASS) and Others v. France (2018),^56^ where the European Court of Human Rights, held that “there had been no violation of Article 8 of the Convention in respect of the complaint of 17 of the individual applicants, finding that the French State had struck a fair balance between the various interests at stake. In particular, taking account of the impact of the whereabouts requirement on the applicants’ private life, the Court nevertheless took the view that the public interest grounds that made it necessary were of particular importance and justified the restrictions imposed on their Article 8 rights. The Court also found that the reduction or removal of the relevant obligations would lead to an increase in the dangers of doping for the health of sports professionals and of all those who practice sports, and would be at odds with the European and international consensus on the need for unannounced testing as part of doping control”.^57^

Sports administrators should understand that, under Article 8 of the ECHR, the concept of private life also covers an individual’s right to develop their identity and to forge friendships and other relationships. This includes a right to participate in essential economic, social, cultural, and leisure activities.^58^ For instance, the European Commission on Human Rights stated that: “*the right to respect for ‘private life’ is the right to privacy, the right to live as far as one wishes, protected from publicity… however, the right to respect for private life does not end there. It comprises also, to a certain degree, the right to establish and develop relationships with other human beings especially in the emotional field, for the development and fulfillment of one’s own personality*”. (X. v. Iceland).^59^ In addition, the International Covenant on Civil and Political Rights^60^ states under Article 17 that “[n]o one shall be subjected to arbitrary or unlawful interference with his privacy, family, home or correspondence (...) Everyone has the right to protection of the law against such interference or attacks.” Similar provisions can also be found under Article 11 (2) and 21 of the American Convention on Human Rights (ACHR) and Article 8 (1) of the ECHR. Hence, sports administrators should encourage athletes and support staff, while in bio-bubbles, to develop relationships with others at the training venues in order to maintain positive mental health, while respecting the COVID-19 implemented measures.

### Promote a peaceful society concerned with the preservation of human dignity

Rebecca Hilsenrath, the Equality and Human Rights Commission Chief Executive, commenting on the human rights implications of restrictions on people’s lives due to the COVID-19 pandemic, has emphasized the need for individuals to have an adequate standard of living:

“We need to find the balance between saving lives from coronavirus, and allowing people the hard won freedoms that are the framework for those lives—such as a right to a private and family life, to freedom of assembly, and to an education. This must go hand in hand with an economic recovery that provides everyone with an adequate standard of living”.^61^

Article 25(1) of the UDHR covers this right: “Everyone has the right to a standard of living adequate for the health and well-being of himself and of his family.” State parties to the International Covenant on Economic, Social and Cultural Rights (ICESCR) also acknowledge the right of everyone to have an adequate standard of living for themselves and their family (Article 11). Such protections are important because there is a strong link between an individual’s surroundings and both their physical and mental health.^62^ For instance, surgical patients assigned to rooms with windows looking out onto natural scenes use fewer potent analgesics, have fewer negative comments in nurses’ notes, and have shorter postoperative hospital stays than patients in similar rooms with windows facing a brick building wall^63^; exercising in natural environments is associated with decreases in anger, confusion, and depression, tension, and greater feelings of energy, positive engagement, and revitalization; and natural forest environments promote better health responses than city environments (e.g., lower blood pressure, pulse rate, and cortisol concentrations).^64^

Two good examples of organizations securing the right of an adequate standard of living come from the Olympic Games and the FIFA World Cup. The Olympic Charter regulates the organization of the Olympic Movement and the celebration of the Olympic Games. It codifies the fundamental principles of Olympism and has a binding force on all members of the Movement. Fundamental principle 2 of the 2007 version of the Charter notes that the “goal of Olympism is to place sport at the service of the harmonious development of man, with a view to promoting a peaceful society concerned with the preservation of human dignity”.^65^ The Charter also establishes the principle of non-discrimination in its principle 5, endorses sustainable development and promotes the idea of leaving a positive legacy from the Olympic Games for the host city and the host country: “Any form of discrimination with regard to a country or a person on grounds of race, religion, politics, gender or otherwise is incompatible with belonging to the Olympic Movement”.^66^ In addition to the respect of the above articles, the Olympic parties and sports administrators are bound by a code of ethics, which restates the obligation to respect the principles of the dignity of the individual and non-discrimination. Similarly, the core values of FIFA are defined as authenticity, unity, performance, and integrity.^67^ Among the general provisions of its statutes, FIFA incorporated a policy of “non-discrimination and stance against racism,”^68^ as well as the promotion of friendly relations in “society for humanitarian objectives”.^69^ Sports administrators must follow Article 3 of the Convention on the Rights of Persons with Disabilities (CRPD) by “providing individual autonomy including the freedom to make one’s own choices, and independence of persons; supporting full and effective participation and inclusion in society without any discrimination; respecting for difference and acceptance of persons with disabilities as part of human diversity and humanity; giving equality of opportunity to all players; facilitating the accessibility; assuring the equality between men and women; taking into consideration for the evolving capacities of children with disabilities and for the right of children with disabilities to preserve their identities”.^70^

### Encourage international cooperation to realize the right to health without discrimination

The ICESCR recognizes the right of everyone to experience “the enjoyment of the highest standard of physical and mental health” (Article 12 (1)). General Comment 14, however, notes that the enjoyment of such a right to health is dependent on environmental circumstances and other rights.^71^ This right to health is also supported in Article 24 of the United Nations Convention on the Rights of the Child,^72^ Article 12 of the Convention on the Elimination of all forms of Discrimination Against Women,^73^ Article 11 of the European Social Charter,^74^ Article 35 of the Charter of Fundamental Rights of the European Union,^75^ Article 10 of the Protocol of San Salvador,^76^ and Article 16 of the Banjul Charter.^77^ Generally, state action is seen as obligatory, as Governments need to take adequate and appropriate measures to pursue the full implementation of and to promote and encourage international cooperation with the view to achieving the full realization of the right to health without discrimination. Hence, sports administrators should follow these measures without any discrimination inside the team to fulfill the right to health to all their players.

### Support quantitatively and qualitatively the adequate right to food

The right to adequate food is protected in many international human rights instruments.^78^ In 2001, the right to food was defined as “[A] human right, inherent in all people, to have regular, permanent and unrestricted access, either directly or by means of financial purchases, to quantitatively and qualitatively adequate and sufficient food to the cultural traditions of people to which it belongs, and which ensures a physical and mental, individual and collective fulfilling and dignified life free of fear”.^79^ Sports administrators should ensure that their athletes and support staff have adequate access to appropriate food and drinks that adhere to basic nutritional principles, such as balance and variety^80^; are in line with nutritional guidelines that aim to prevent chronic diseases^81^; take into consideration medical conditions^82^; and are culturally and religiously sensitive.^83^ Therefore, nutrition is the foundation of performance enhancement, where if missed, athletes cannot compete to their full potential.^84^ For instance, as water is a key nutritional component for athletes, coaches should not restrict it as punishment within a training, as this could lead to a reduction in performance and possible serious health consequences.^85^ In addition, athlete’s special nutritional requirements also need to be catered for.^86^

### Respect for legal duties toward the athletes

In most contact sport, players are prone to injuries, where former professional athletes are now suffering life-limiting conditions following retirement from sport. For instance, lawyers for more than 185 players are suing rugby union’s governing bodies [World Rugby, the Rugby Football Union (RFU) and the Welsh Rugby Union (WRU)] for the negligence of failing to “protect players from permanent injury”, claiming that playing the sport had caused brain damage.^87^ The most recent of these is former England Rugby Union World Cup winner, Steve Thompson and seven former players who are considering a claim of negligence against the sporting governing body.^88^

Administrators and coaches owe a duty of care to participants. Where a sporting body assumes the role of a rule maker and, dangers abound, there is, arguably, a risk of a duty being owed by the organization to each participant.^89^ For example, it is often the responsibility of an official (or the relevant council if they have responsibility for the field of play) to determine the playability of the field, including for training purposes. If they fail to ensure that the ground is in a safe condition to train or play on, the official may be in breach of their duty of care toward the athletes (plaintiff)^90^ (see Wagga Wagga City Council v Mark Sutton [2000] NSWCA 34). In addition, it is certainly the responsibility of officials to set safety standards for competition, where coaches need to be concerned about the welfare of their players and the maintenance of athletic equipment and facilities^91^. For instance, the case of *Dyke v. British Columbia Amateur Softball Association*^92^ demonstrates an example of a standard of care assessment, in the context of a scorekeeper at a softball game who suffered a head injury after being hit by a foul ball. The scorekeeper had been standing in an unprotected area and not in the dugout because it was flooded. No breach of the standard of care was found because the occupier provided safe alternative locations for the scorekeeper to stand in.^93^ The Court of Appeal confirmed the trial judge’s definition of the standard of care as follows:

*With respect to being struck by foul balls the standard of care for occupiers is to provide, adequate fencing in order to protect those persons located in ‘danger zones’, those areas where the risk of being struck would otherwise be unreasonably high.*

In assessing the requisite standard of care for the occupier with respect to adequate fencing in the above case, the reasonable standard of protection was largely determined with reference to industry standards. Accordingly, there is no obligation to provide absolute protection in facilities designed for the viewing of a particular sport; the protection only needs to be reasonable.

However, the following list of legal duties of a coach is adapted from the Coaching Youth Sports website and is recommended for coaches and administrators.^94^

1. Conducting practices and games in a safe physical environment.
2. Use of current knowledge of proper skills and methods of instruction.
3. Use of safe and appropriate equipment.
4. Proper short- and long-term planning.
5. Proper matching of athletes in practices and games by size, experience and ability.
6. Provision of adequate supervision of athletes.
7. Provide warnings to parents and athletes of risks inherent in sport participation.
8. Sensitivity to the health and well-being of athletes under a coach’s care.
9. Provision of appropriate emergency care.

Importantly, sporting organizations, coaches, and officials should ensure that adequate first aid and emergency services are available to respond to any injury.^95^ The more dangerous the sport, the more comprehensive the service should be, particularly if there is little difficulty in terms of people power or cost to minimize the effects of a known sporting danger, where coaches are often the first responders when immediate medical care is not provided by an allied healthcare professional.^96^

## Psychological advice sports administrators should provide athletes and support staff

Unless athletes, coaches, and support staff engage in functional behaviors, they risk experiencing negative psychological effects such as anger, burnout, confusion, depression, and loneliness, while living in bio-bubbles and quarantine procedures. To reduce the likelihood of experiencing such negative psychological effects, we shall now provide ten suggestions that sports administrators should consider implementing immediately. These 10 suggestions can be implemented in consultation with independent experts knowledgeable about local conditions, cultures, government rules and regulatory support.

### Emphasize flourishing mental health

Too many sports organizations, even at the international level, provide no mental skills training for their athletes and staff, little in the way of preventative mental illness training, and reluctantly only utilize mental skills experts once a problem has arisen.^97^ Such psychological help tends to; unfortunately, focus on the negative end of personality continuums, such as introversion, neuroticism, and pessimism. There is an urgent need to move away from such a negative orientation. The absence of mental illness should not be the agenda. The emphasis needs to be on the positive end of personality continuums, especially the presence of flourishing mental health.^98^ Flourishing is “the experience of life going well… a combination of feeling good and functioning effectively… synonymous with a high level of mental well-being, and it epitomizes mental health”.^99^ Flourishing encompasses a wide range of positive psychological constructs, such as competence, emotional stability, engagement, meaning, optimism, positive emotion, positive relationships, resilience, self-esteem, and vitality^100^, and can be improved with psychological and lifestyle strategies.^101^ Focusing on enhancing flourishing will have the added advantage of not encountering the deeply discrediting effects of the perceived stigma associated with treatment for mental illness,^102^ especially in the athletic world,^103^ and the resulting frequent non-completion of recommended mental illness treatment.^104^

### Emphasize training (not counseling/therapy)

Psychology is usually viewed by the public as “a kind of ‘medical practice’ for people with ‘sick souls’ or ‘sick minds’”.^105^ As a result, mentally healthy people tend to miss what psychology has to offer.^106^ Hence, sports administrators should not advocate traditional psychological counseling/therapy as the fall-back modus operandi for their athletes, coaches, and support staff. Instead, it should be part of every athlete’s training regimen, akin to physical training. This training could involve mental skills experts teaching things like the importance of, and techniques to increase, conscientiousness,^107^ mental toughness (and its subcomponents: confidence and hardiness),^108^ and the five NOISE personality styles (and two associated techniques: NOISE affirmation and Thriving Habit).^109^ An added advantage of such mental skills training is that it parallels attempts to achieve flourishing mental health.

### Emphasize prevention of mental issues with mental skills training and life skills training

Sports administrators, however, should not altogether dismiss mental illness, especially as an estimated 25% of the worldwide population is affected by a mental or behavioral disorder at some time during their life,^110^ research with university-aged athletes reports 18 percent have had a previous mental illness diagnosis,^111^ and it appears that mental illness is underdiagnosed in athletes.^112^ Instead, they should follow some recent trends in clinical care^113^ and mental health care^114^ that have seen a change from emphasizing cure to advocating prevention of mental disorders and promotion of positive mental health.^115^ Sports administrators would do well to remember that: “The great public health success stories of the past century are largely stories of prevention. From sanitation to vaccines to smoking cessation to the use of statins, we have proven much more successful at pre-empting disease than curing it”.^116^ Hence, future pandemic periods, when most international athletes and support staff have more time available for such training, should be viewed as a fantastic opportunity for athletes and staff. In particular, an education model should be used which combines mental skills training to improve performance^117^ with life skills training to improve mental health,^118^ so that athletes and support staff live more proactive, positive, and healthy lives.^119^

### Encourage athletes/staff to research what techniques others have found helpful

Sports administrators can help athletes and staff effectively combine preventative mental skills and life skills training, with a flourishing mental health focus, by providing articles about individuals in similar situations, such as solitary confinement. A pertinent article, published by Neilson,^120^ details how a prisoner felt free in solitary confinement and left prison a better man. Likewise, Kremer and Hammond discussed what techniques prisoners confined in solitary have found beneficial.^121^ Conclusions created by reading those two articles can be profitably contrasted with articles focusing on what others, not using such techniques, have experienced. For example, J. Wesley Boyd argues, “Let’s call it for what it is: Placing prisoners in solitary confinement is tantamount to torture and it needs to stop”.^122^ Finally, they could read an article,^123^ which provides suggestions parents can provide their children during COVID-induced lockdowns. These suggestions (e.g., limit exposure to news, use a consistent routine filled with activity, ensure a consistent time for going to sleep) are equally relevant for adults.

### Encourage athletes/staff to use strategic planning techniques with a personal focus

Tom Harrison, the England and Wales Cricket Board’s Chief Executive Officer has warned, “You want players turning up in these ‘most important series’ feeling fantastic about the opportunity of playing for their country… They are not going to be able to achieve that if they have forgotten the reasons why they play”.^124^ Hence, every athlete and support staff member, especially in lockdowns and bio-bubbles, should have a personal mission, vision, and purpose statement, along with values (and their definitions), a motto, and an elevator conversation^125^ and not just team strategic statements. Athletes and support staff should be taught how to create their mission, vision, purpose statements, and then have them printed and laminated on cards that they can easily travel with and thus see daily. Likewise, they should be challenged to accurately determine their most cherished values and then helped to define them. They may like to create a motto that encapsulates their mission, vision, purpose, and values. Then they should be encouraged to produce an elevator communication that they can say to themselves every day to remind themselves why they are pursuing their sports goals and thus willingly living in a bubble. Finally, they should be taught how to incorporate some or all of their mission, vision, and purpose statements, values, mottos, and elevator conversations into the daily practice of the 5-minute Thriving Habit technique.^126^ These suggestions are in line with the TRIPE approach for improving concentration in sport,^127^ which suggests that athletes and staff should remain Positive and control their Environment. By reading and thinking about their mission, vision, purpose, values, motto, and elevator communication, the athletes are proactively controlling their thinking and emotions.

### Urge participation in hobbies

Sports administrators should encourage athletes and support staff to either start a new hobby or put more time and effort into an already-learnt hobby, as adults just “vegetating” report little satisfaction, whereas individuals actively involved in engaging their skills, are happier.^128^ For instance, Massimini and Carli note 39% of people watching television feel apathetic compared with only 4% engaged in arts and hobbies.^129^ Athletes and staff should also be advised not to fill in their extra time with additional work, as Li et al. have shown that individuals who work more than 60 h per week have poorer mental health and more depression compared with individuals working 40 or fewer hours per week.^130^ They also found that those who had hobbies had less depression and more positive mental well-being. There is no need for people in quarantine to become more inactive, irritable, passive, sad, and weak if they are actively pursuing proactive or enjoyable past-times. Part of this time can be spent proactively planning for life after sport. This will aid future mental health as Trento has discovered that a lack of planning for life after sport results in a negative retirement transition and decreased quality of life in retirement, whereas athletes who explored internships and hobbies outside of sport experience more perceived wellness.^131^

### Encourage the deletion of some social media accounts

As international athletes and support staff have to spend more time by themselves during the pandemic, it’s easier for them to be distracted and unfortunately impacted by negative feedback from others via online devices and platforms. Hence, they would be well advised to consider deleting social media accounts (e.g., Facebook and Twitter accounts) to prevent or decrease unnecessary negative feedback and opportunities for social comparison, especially from unsolicited, unknown, or anonymous individuals. Athletes and staff would also profit from learning the research findings correlating internet use with happiness. Some studies show no relationship,^132^ other research suggests a negative relationship,^133^ but no study indicates happy people use the internet more frequently than unhappy individuals.^134^ There are studies, however, that have found both internet use and internet addiction are related to depression.^135^ Encouragingly, a study of university students found that those who limited Facebook, Instagram, and Snapchat use to 10 min, per platform, per day, had significant reductions in loneliness and depression compared with those who continued their usual social media use.^136^

### Support communication flexibility and negative issues monitoring

Csikszentmihalyi and Hunter asked individuals to carry a pager and write down how happy they were when the pager was activated.^137^ The participants were happiest when they were with friends and least happy when alone. Likewise, Diener and Seligman have observed a correlation between happiness and participation in romantic and social relationships.^138^ Hence, bubble life and quarantines pose serious impediments to happiness. Those in isolation will benefit substantially if their friends and/or family members are flexible in their modes of engagement. Phone calls, video calls, and Telepathy (a medium that allows you to watch television with others online) should be recommended. Setting up a regular day and time to contact friends and family is advocated, along with scheduling virtual get-togethers on online platforms. However, athletes and staff should monitor the amount of time spent talking about negative issues. Caution is also needed concerning how much pandemic information is viewed, as Li et al. have found that individuals who overindulge in epidemic information, especially those who are conscious about epidemic information for more than 3 h per day, are more likely to be depressed and anxious.^139^ Avoiding all COVID-19 information, however, is not a good strategy, as those in quarantine who had a low concern for epidemic information were more likely to be depressed.

Li et al. concluded: “It would be worthwhile to provide online or smart phone-based psycho education about the COVID-19, promote mental wellness and initiate psychological intervention.^140^ It is noteworthy that the interventions should be implemented to help people to limit the time they spend on social media and to obtain accurate information related to the epidemic of the COVID-19 from authoritative and authentic resource to prevent psychological problems.^141^”

### Encourage the use of time management techniques and daily routines

Administrators may like to suggest athletes and staff use one or more time management techniques while in lockdowns and bio-bubbles. Athletes and staff may choose to create a “Not-to-do list” to avoid unnecessary tasks.^142^ Likewise, they can make a table stating all the activities they want to perform. Then at the end of the night, or as they finish an activity, they can make themselves more accountable by recording which activities they performed. This approach will probably give them a heightened feeling of competence and help them stay focused on what matters. Individuals may prefer to use one of the many digital Personal Information Management (PIM) applications, handheld personal digital assistants (PDAs), or web-based task list applications available. However, they should be advised not to rigidly utilize such approaches, as this may lead to non-ideal feelings that they must complete everything on their To-Do list and feeling their life is being run by items stored on a list.^143^ These time management techniques can be used as a basis to help create daily routines. Such routines can be very effective because they reduce or totally remove the need for decision-making and therefore remove anxiety, indecision, and procrastination.^144^

### Protect athletes and staff from unnecessary media questioning

Sports administrators should shield international athletes, coaches, and support staff from the media if they need time away from sport. The onus should be for sports administrators to inform the media of such decisions. Athletes, in particular, should not have to discuss such issues with the media. Administrators and the media should take heed of tennis player Naomi Osaka’s opinion, “It has become apparent to me that literally everyone either suffers from issues related to their mental health or knows someone who does… Perhaps we should give athletes the right to take a mental break from media scrutiny”.^145^ Ideally, journalists should hear such requests to be more empathic and less demanding of athletes.

## Conclusion

Guaranteeing human rights for every athlete, coach, and support staff is a substantial challenge for every sports administrator. Nevertheless, if sports administrators focus on respecting human rights, especially the right to private and family life, an adequate standard of living, health, and food, they will decrease the likelihood of bio-bubble and quarantine breaches and/or violations. In addition, their decisions should be in line with and foster values of fairness, integrity, respect, and responsibility.^146^

Sports administrators would do well to heed the words of the Secretary-General of the Council of Europe that emphasize good practices and positive support: “While the virus is resulting in the tragic loss of life, we must nonetheless prevent it from destroying our way of life—our understanding of who we are, what we value, and the rights to which every European is entitled”.^147^ Likewise, sports administrators should follow the call-to-action of Antonio Guterres, the United Nations Secretary General, by making human rights central to the COVID-19 response and recovery: “This is not a time to neglect human rights; it is a time when, more than ever, human rights are needed to navigate this crisis in a way that will allow us, as soon as possible, to focus again on achieving equitable sustainable development and sustaining peace”.^148^ Hence, in future pandemics, all teams should have an orientation session before any tournament to ensure that the human rights of players, coaches, and support staff are being respected.

Sports administrators are encouraged to suggest to their athletes and support staff ten techniques they can utilize when in bio-bubbles or quarantines: (1) Emphasize the creation of flourishing mental health via mental skills training (and less focus on preventing mental illness); (2) Emphasize training (not psychological counseling/therapy) as the main mental skills helping approach; (3) Emphasize regular, structured, and comprehensive mental skills and life skills training with an expert mental skills trainer (or sport psychologist); (4) Encourage athletes and staff to research what techniques others in similar situations have found helpful; (5) Ensure athletes and staff are taught how to create their own mission, vision, purpose, values, motto, and elevator communication; (6) Encourage athletes and staff to either start a new hobby or put more time and effort into already-learnt hobbies; (7) Encourage athletes and staff to consider deleting some social media accounts (e.g., Facebook and Twitter accounts); (8) Encourage athletes and staff to be open to using a variety of communication devices to engage with friends and family (e.g., phone calls, video calls, and Teleparty) while monitoring the amount of time they spend discussing negative issues; (9) Encourage the use of time management techniques and daily routines; and (10) Shield athletes/staff from the media if they need time away from sport.

This article has illuminated an urgent need for more research into online communication. While the need for social interaction in bio-bubbles via electronic devices needs to be encouraged, current research is indicating possible harmful psychological effects of social media use. Hence, researchers should determine (1) whether social media time limits result in significant psychological benefits and (2) the most effective ways to monitor time spent talking about negative issues on social media. In addition, sports administrators will benefit from two post-COVID-19 reviews. First, legal and psychological researchers should retrospectively examine actions during the pandemic and ascertain whether an optimal balance between protecting health and liberty was achieved. Second, this article can serve as a guideline for reviews focusing on preparation for future pandemics or similar crises.

When creating, implementing, and enforcing bio-bubble policies and procedures, sports administrators must balance protecting the health of their athletes, staff, and the wider population (by preventing the spread of the COVID-19 disease) with not restricting anyone’s liberty and unalienable rights to pursue happiness. For instance, athletes should be able to opt out of the training environment at any time without any resulting discrimination not associated with the potential natural competitive impact resulting from any loss of training time.^149^ Our central proposition is that sports administrators should, in an attempt to find this balance, be constantly focused on flourishing mental health and human rights. As flourishing can only be attained through one’s efforts and requires a life with others,^150^ bio-bubble and quarantine policies must facilitate every individual’s ability to take charge of their own lives so that they can achieve this desirable state of feeling good combined with functioning effectively. A human rights-sensitive approach to policies will increase the likelihood of dignified lives and the enjoyment of basic freedoms. Impeding such fundamental human rights, however, is unacceptable, because as South African President Nelson Mandela has noted: “To deny people their human rights is to challenge their very humanity”.^151^

## Footnotes

1 World Health Organization (n.d) Coronavirus (COVID-19) Dashboard. https://covid19.who.int/, Accessed 22 February 2023.

2 Campbell et al. (2020).

3 He and Harris (2020).

4 Siche (2020).

5 Delardas et al., (2022).

6 He and Harris (2020).

7 Harper et al. (2020).

8 Pokhrel and Chhetri (2021).

9 Sarkodie and Owusu (2021).

10 McCurry and Ingle (2020).

11 Decent (2020).

12 Boren and Bonesteel (2020).

13 Clarey (2020).

14 Aschburner (2020).

15 Feinsand (2020).

16 Da Silva (2020).

17 Premier League (2020).

18 Reuters Staff (2020).

19 Espn (2020).

20 Brito de Souza et al. (2021).

21 Mulvenney (2020).

22 Stein (2020).

23 Nxumalo (2020).

24 Rigal (2021).

25 Gollapudi (2021).

26 Brooks et al. (2020).

27 Wang et al. (2021).

28 Robertson et al. (2004).

29 Liu et al. (2012).

30 Bansal and Sheriff (2021).

31 ibid.

32 AFP. (2021).

33 Nakrani and Paley (2021).

34 ibid.

35 Gollapudi (2021).

36 Gollapudi (2021).

37 Pakistan Cricket Board (2021).

38 ibid.

39 Futterman (2021).

40 Campbell et al. (2020).

41 United Nations (2014).

42 Harrington (2020).

43 Rogers (2020).

44 Woodcock (2006).

45 United Nations (1948).

46 European Convention on Human Rights (1950).

47 Office of the High Commissioner for Human Rights (1966).

48 United Nations treaties Collection (1979).

49 African Commission on Human and Peoples’ Rights (1986).

50 Newman and Vasak (1982).

51 Human Rights Commission (2004).

52 Human Rights Commission (1982).

53 World Health Organization team (2020).

54 Gerstell (2020).

55 Dzehtsiarou (2020).

56 European Court on Human Rights, (2018).

57 European Court on Human Rights, Sport and the European Convention on Human Rights (2023).

58 Equality and Human Rights Commission (2021)

59 Iceland Human Rights Centre (n.d).

60 Office of the High Commissioner for Human Rights (1966).

61 Equality and Human Rights Commission (2020).

62 Seymour (2016).

63 Ulrich (1984).

64 Park et al. (2010).

65 The Olympic Charter (2011).

66 ibid.

67 Tomlinson (2014).

68 FIFA Statutes (2018a, b).

69 FIFA Statutes (2018a, b).

70 United Nations Convention on the Rights of Persons with Disabilities (2006).

71 The ICESCR states under “General Comment No. 14 (2000) Right to the highest attainable standard of health (Art. 12) U.N. Doc. E/C.12/2000/4 (11 August 2000) para.11 (emphasis added): “an inclusive right extending not only to timely and appropriate health care but also to the underlying determinants of health, such as access to safe and potable water and adequate sanitation, an adequate supply of safe food, nutrition and housing, healthy occupational and environmental conditions, and access to health-related education and information, including on sexual and reproductive health. A further important aspect is the participation of the population in all health-related decision-making at the community, national and international level.”

72 United Nations Convention on the Rights of the Child (1989).

73 United Nations Convention on the Elimination of All Forms of Discrimination against Women (1979).

74 Council of Europe (2015).

75 European Commission (2012).

76 Protocol Of San Salvador (1988).

77 African Commission on Human and Peoples’ Rights (1986).

78 The ICESCR recognizes under Article 11 (1),”the right of everyone to an adequate standard of living for himself and his family, including adequate food, clothing and housing and to the continuous improvement of living conditions” while pursuant to Article 11 (2) they recognize that urgent steps may be necessary to ensure “the fundamental right to freedom from hunger and malnutrition”. The right to food has been included under Article 25 of UDHR; Article 12(2), Articles 24 (2) (c) and 27 of CEDAW.

79 “Report by the Special Rapporteur Jean Ziegler on the right to food to the Commission on Human Rights 57th session” U.N. Doc. E/CN.4/2001/53 (7 February 2001) p. 8 para14.

80 Insel et al. (2017).

81 Krauss et al. (1996).

82 Coulston et al. (2017).

83 Kittler et al. (2016).

84 Scott et al. (2011).

85 Fuller and Drawer (2004).

86 Benardot (2020).

87 Rugby Union (2022).

88 McLaughlin (2020).

89 The Queensland Law Handbook (2021).

90 ibid

91 Scott et al. (2011).

92 Court of Appeal (British Columbia). Dyke v. Amateur Softball Assoc. (2008).

93 Dolden Wallace Folick Llp (2012).

94 Rich Engelhorn (n.d).

95 The Queensland Law Handbook (2021).

96 Bradford Strand et al. (2019).

97 Haikali (2021).

98 Diener et al. (2009), Seligman (2011).

99 Huppert and So (2011).

100 ibid.

101 Przybylko et al. (2021).

102 Ahmedani (2011).

103 Schwenk (2000).

104 Corrigan (2004).

105 Maddux (2005).

106 Keyes (2007).

107 Mirwah (2021).

108 McInman (2021a).

109 ibid.

110 World Health Organization (2001).

111 Van Slingerland et al. (2018).

112 Schwenk (2000).

113 Gérvas et al. (2008).

114 Carter et al. (2021).

115 Fusar-Poli et al. (2019).

116 Insel and Scolnick (2006).

117 McInman (2021a).

118 Fogaca (2021).

119 Lore (2011).

120 Neilson (2016).

121 Kremer and Hammond (2013).

122 Katznelson and Boyd (2018).

123 Singh et al (2020).

124 Gollapudi (2021).

125 McInman (2021a), McInman (2021b).

126 McInman (2021a).

127 ibid.

128 Csikszentmihalyi and LeFevre (1989).

129 Massimini and Carli (1988).

130 Li et al. (2019).

131 Trento (2020).

132 Gross (2004). Gross et al. (2002).

133 Veenhoven and Vogelaar (2019).

134 McInman (2021a).

135 Banjanin et al. (2015).

136 Hunt et al. (2018).

137 Csikszentmihalyi and Hunter (2003).

138 Diener and Seligman (2002).

139 Li et al (2021).

140 Sidi (2020).

141 Woon et al. (2020).

142 Morgenstern (2004).

143 Hendrickson (2003).

144 Robards (2014).

145 Osaka (2021).

146 Hanson and Savage (2012).

147 Burić (2020).

148 Guterres (2020).

149 Gunawardena (2020).

150 Rasmussen (1999).

151 Hadden (2019).
